# Functional polymorphisms in *NR3C1* are associated with gastric cancer risk in Chinese population

**DOI:** 10.18632/oncotarget.22172

**Published:** 2017-10-27

**Authors:** Yayun Gu, Bin Deng, Jing Kong, Caiwang Yan, Tongtong Huang, Jianshui Yang, Yan Wang, Tianpei Wang, Qi Qi, Guangfu Jin, Jiangbo Du, Yanbing Ding, Li Liu

**Affiliations:** ^1^ Department of Epidemiology and Biostatistics, School of Public Health, Nanjing Medical University, Nanjing, China; ^2^ Jiangsu Key Lab of Cancer Biomarkers, Prevention and Treatment, Collaborative Innovation Center for Cancer Medicine, Nanjing Medical University, Nanjing, China; ^3^ Department of Gastroenterology, Affiliated Hospital of Yangzhou University, Yangzhou, China; ^4^ Digestive Endoscopy Center, The First Affiliated Hospital of Nanjing Medical University and Jiangsu Province Hospital, Nanjing, China

**Keywords:** polymorphism, NR3C1, gastric cancer, Chinese population

## Abstract

Recently promoter of *NR3C1* has been found to be high methylated in gastric cancer tissues which might be involved in the initiation of gastric carcinoma development. To test whether the variants in *NR3C1* could modify the risk of gastric cancer, we evaluated the association between four SNPs (rs6194, rs12521436, rs33388 and rs4912913) in *NR3C1* and gastric cancer risk in a case-control study with 1,113 gastric cancer cases and 1,848 cancer-free controls in a Chinese population. We found a significant association between rs4912913 and gastric cancer risk (OR=1.18, 95%CI=1.05-1.33, *P*=5.49×10^−3^). We also observed that the A-allele of rs12521436 and rs33388 were significantly associated with a decreased risk of gastric cancer (OR=0.84, 95%CI=0.76-0.94, *P*=2.78×10^−3^; OR=0.85, 95%CI=0.75-0.97; *P*=0.018). Finally, we made a joint effect analysis of rs12521436, rs33388 and rs4912913 on risk of gastric cancer (*P_Trend_*=2.83×10^−5^). These findings indicate that the variants rs4912913, rs33388 and rs12521436 of *NR3C1* may contribute to gastric cancer susceptibility.

## INTRODUCTION

Gastric cancer (GC) is the fourth most common cancer in the world and the second leading cause of death due to cancer, reflecting the overall 5 year survival rate (5YSR) of 24% [1–3]. Chen *et al*. reported that more than 70% of cases (679,100 cases) occur in China (477,700 in men, 201,400 in women) [4]. Currently, many epidemiologic studies have demonstrated that gastric cancer has a multifactorial etiology and is co-modulated by different factors including Helicobacter pylori infection [5], life style [6], socioeconomic status [6], and environmental factors [5, 7–10]. However, only few individuals exposed to these risk factors ultimately develop gastric cancer, suggesting genetic factors may influence susceptibility to gastric cancer development [11–13]. Therefore, identification of genetic biomarkers significantly related to risk of gastric cancer in candidate genes is essential for better gastric prevention. Genome-wide association studies (GWAS) have discovered a few significant loci associated with gastric cancer, including 1q22, 5p13.1, 10q23 and 8q24.3 [14–16]. However, these results could only explain a fraction of the heritability of the gastric cancer because GWAS often focus on the most significant associations. Those loci with relative moderate signal might be ignored. Therefore, candidate gene based association study is still crucial in identifying those vital genes in carcinogenesis.

Previous studies showed that glucocorticoid hormone was involved in development of various cancers. For example, impairment of glucocorticoid hormone-STAT5 signaling in different experimental models was reported to be correlated with hepatocellular carcinoma (HCC) [17]. Additionally, an operational glucocorticoid system in breast tissue was identified to influence breast cancer development [18]. As a main regulator of glucocorticoid hormone effects, nuclear receptor subfamily 3 group C member 1 (NR3C1) is a member of the nuclear hormone receptor super family of ligand-activated transcription factors [19], leading to altered gene expression in target cells and tissues [20–23]. In previous studies, *NR3C1* was identified as epigenetically deregulated gene in gastrointestinal tumorigenesis [24, 25]. In addition, glucocorticoid receptor was overexpressed in malignant adrenocortical tumors [26], and activation of glucocorticoid receptor was associated with poor prognosis in estrogen receptor-negative breast cancer [27]. Recently, it is noteworthy that expression of *NR3C1* was found to be associated with liver metastasis of gastric cancer [28] and it was high methylated in gastric cancer which might play a key role in the initiation and progression of gastric carcinoma development [29]. More interestingly, studies found that glucocorticoids may act as natural defensive factors in maintaining the integrity of the gastric mucosa during nonsteroidal anti-inflammatory drug (NSAID) therapy and might operate to attenuate NSAID-induced gastropathy [30].

Refer to the above actuality, we hypothesized that genetic variants in *NR3C1* may play an important role in gastric cancer susceptibility. To test this hypothesis, we conducted a case–control study including 1,113 gastric cancer cases and 1,848 controls to investigate the association of functional polymorphisms in *NR3C1* with risk of gastric cancer in a Chinese population.

## RESULTS

The distribution of selected characteristics between 1,113 GC cases and the 1,848 cancer-free controls are summarized in Supplementary Table 2. There were no significant differences between cases and controls in the distribution of age, sex, and drinking status (*P*=0.135, 0.115 and 0.709, respectively).

Genotyping call rates were all 100% for rs4912913, rs6194, rs12521436 and rs33388. The observed genotype frequencies for these variants were in Hardy-Weinberg equilibrium (HWE) among the controls (*P*=0.62 for rs4912913, *P*=0.59 for rs6194, *P*=0.11 for rs12521436 and *P*=0.18 for rs33388) (Supplementary Table 1). In the additive model, we found a significant association between rs4912913 and gastric cancer risk (OR=1.18, 95%CI=1.05-1.33, *P*=5.49×10^−3^). We also observed that the A-allele of rs12521436 and rs33388 were significantly associated with a decreased risk of gastric cancer (OR=0.84, 95%CI=0.76-0.94, *P*=2.78×10^−3^; OR=0.85, 95%CI=0.75-0.97; *P*=0.018) (Table 1).

**Table 1:** Association results of four SNPs in *NR3C1* with gastric cancer risk

SNP	Cases	Controls	OR (95%CI)^a^	*P* value^a^
	1113 (%)	1848 (%)		
**rs4912913**				
AA	485(43.58)	890(48.16)	1.00	
AG	499(44.83)	792(42.86)	1.15(0.97-1.35)	0.097
GG	129(11.59)	166(8.98)	1.44(1.10-1.88)	7.34×10^−3^
AG/GG			1.20(1.03-1.40)	0.023
Additive model			1.18(1.05-1.33)	5.49×10^−3^
**rs6194**				
GG	981(88.14)	1593(86.20)	1.00	
GA	126(11.32)	248(13.42)	0.86(0.67-1.09)	0.203
AA	6(0.54)	7(0.38)	1.74(0.56-5.47)	0.340
AG/AA			0.88(0.69-1.11)	0.280
Additive model			0.91(0.73-1.14)	0.399
**rs12521436**				
GG	395(35.49)	575(31.11)	1.00	
GA	542(48.70)	881(47.67)	0.92(0.77-1.09)	0.339
AA	176(15.81)	392(21.21)	0.69(0.55-0.87)	1.55×10^−3^
AG/AA			0.85(0.72-1.00)	0.051
Additive model			0.84(0.76-0.94)	2.78×10^−3^
**rs33388**				
TT	706(63.43)	1055(57.09)	1.00	
TA	347(31.18)	697(37.72)	0.75(0.63-0.88)	6.37×10^−4^
AA	60(5.39)	96(5.19)	0.99(0.70-1.41)	0.958
TA/AA			0.78(0.66-0.91)	1.82×10^−3^
Additive model			0.85(0.75-0.97)	0.018

^a^ Derived from logistic regression with an adjustment for age, sex, smoking, drinking status and the top ten principal components.

Furthermore, subgroup analyses stratified by age, sex, smoking and drinking status were conducted for the associations of these 3 significant single nucleotide polymorphisms (SNPs) with gastric cancer risk (Supplementary Table 3). The rs33388-A allele was associated with a significantly decreased risk of gastric cancer in age (<60), female and no-drinking status. In age (≥60), female, no-smoking and no-drinking status, the A allele of rs12521436 was associated with a significantly decreased risk of gastric cancer, while rs4912913-G allele was associated with a significantly increased risk of gastric cancer. However, there was heterogeneity in the age subgroups of SNPs rs12521436 and rs4912913 and in the sex subgroup of rs33388, while no obvious evidences of significant associations between these SNPs among other subgroups, and *P* for heterogeneity test in different status was greater than 0.05.

In addition, as shown in Table 2, we combined these three loci on an allele to detect the potential joint effects and found that the risk of GC significantly increased with the number of risk allele increasing (*P_Trend_* =2.83×10^−5^). Individuals with 4∼5 risk alleles had a 1.52-fold (95% CI: 1.14-2.04) increased risk of GC, with 6 risk alleles had a 2.6-fold (95% CI: 1.73-3.9) increased risk of GC, compared with those having 0-1 of the risk alleles. Finally, we made a haplotype analysis and found that haplotypes GG was significantly associated with increased risk of gastric cancer (OR=1.23, 95%CI=1.08-1.40, *P*=1.00×10^−3^) as compared with the most common haplotype AA (Table 3). In age (≥60), we observed that the haplotype GG of rs12521436 and rs4912913 were significantly associated with increased risk of gastric cancer (OR=1.50, 95%CI=1.14-1.98, *P*=3.39×10^−3^; OR=1.74, 95%CI=1.21-2.49; *P*=2.80×10^−3^), additionally, the *P* for multiplicative interaction of rs12521436 and rs4912913 with age on gastric cancer risk were all less than 0.05 (*P*=0.018 and 0.013, respectively) (Supplementary Table 4).

**Table 2:** Joint-risk effect of rs12521436, rs33388 and rs4912913 on gastric cancer risk

Risk allele number^a^	Cases	Controls	Adjusted OR^b^	*P* value^b^
0-1	84(7.55)	208(11.26)	1.00	
2-3	482(43.31)	848(45.89)	1.40(1.05-1.87)	0.022
4-5	456(40.97)	705(38.15)	1.52(1.14-2.04)	4.63×10^−3^
6	91(8.18)	87(4.71)	2.60(1.73-3.90)	3.91×10^−6^
Trend				2.83×10^−5^

^a^ rs12521436_G, rs33388_T and rs4912913_G were assumed as risk alleles;

^b^ Derived from logistic regression adjusted for age, sex, smoking, drinking status and the top ten principal components.

**Table 3:** Association analysis between haplotypes of *NR3C1* and gastric cancer susceptibility

Haplotype^a^	All subjects	Case	Control	OR(95% CI)	*P*^b^
AA	0.432	0.401	0.450	1.00	
GA	0.251	0.259	0.246	1.12(0.98-1.29)	0.096
GG	0.317	0.339	0.303	1.23(1.08-1.40)	1.00×10^−3^

^a^ Haplotype was presented with the allele order of rs12521436 and rs4912913;

^b^ Derived from logistic regression with an adjustment for age, sex, smoking, drinking status and the top ten principal components.

**Figure 1:**
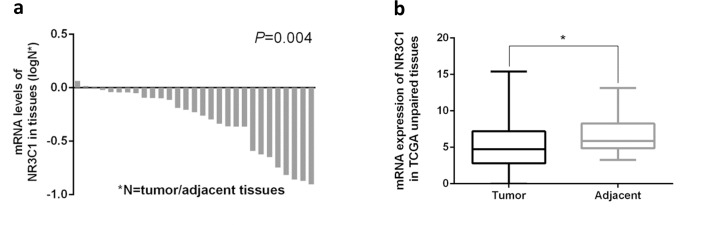
Expression of *NR3C1* in tumor/adjacent gastric tissues and correlation of *NR3C1* mRNA expression levels with gastric cancer survival **(a, b)** Normalized mRNA expression of *NR3C1* was downloaded from TCGA database (https://confluence.broadinstitute.org/display/GDAC/Dashboard-Stddata). The *NR3C1* mRNA expression in tumor was strongly lower than adjacent tissues. *P* value was determined using a two-tailed t-test.

According to the web-based SNP analysis tool HaploRegV3, the SNPs (rs10041520, rs4582314 and rs4634384) have strong linkage disequilibrium (r^2^>0.8) with rs33388 are located on the promoter of *NR3C1*, which were strongly modified by histone H3K27Ac, and might result in aberrant transcription of *NR3C1* [31] (Supplementary Figure 1 and Supplementary Table 5). Meanwhile, Westra *et al*. found that SNPs have strong linkage disequilibrium with the rs33388 in ENCODE project presented cis-eQTL with *NR3C1* [32] (Table 4), and the mRNA expression of *NR3C1* in tumor was significantly lower than adjacent tissues in TCGA database (Figure 1). The above results suggest that these functional SNPs might influence gastric cancer development through the regulation of *NR3C1* expression.

**Table 4:** Expression quantitative trait loci (eQTL) analyses of SNPs that have strong linkage disequilibrium with rs33388 with *NR3C1* mRNA expression levels in whole blood

SNP	Chr	Positions	Alleles	r^2^	cis-eQTL gene	Tissue	*P* value^a^
rs258763	5	143272796	T/A	0.95	NR3C1	Whole blood	0.003
rs853175	5	143257225	T/C	0.86	NR3C1	Whole blood	0.003
rs17287745	5	143275450	A/G	0.56	NR3C1	Whole blood	6.25×10^−4^
rs17209237	5	143277647	A/G	0.56	NR3C1	Whole blood	0.003
rs10482682	5	143299832	A/G	0.43	NR3C1	Whole blood	0.002
rs4912903	5	143262956	C/T	0.38	NR3C1	Whole blood	8.96×10^−5^
rs2398587	5	143240961	C/T	0.30	NR3C1	Whole blood	0.002

^a^ The *p* value of eQTL results were referred to study of Westra *et al* (PMID: 24013639).

## DISCUSSION

Our study provided genetic evidence that *NR3C1* might be involved in the development of gastric cancer and further highlight the importance of *NR3C1* in tumorigenesis. We found that rs12521436, rs33388 and rs4912913 in *NR3C1* were significantly associated with gastric cancer risk. We also observed a significant joint effect of rs12521436, rs33388 and rs4912913 on risk of GC.

The variants rs33388 and rs12521436 are located in intron and 2.5kb far from 5′ of *NR3C1* respectively. The motifs of these two SNPs were predicted to be transcription factor binding sites (TFBS) in SNPinfo database. Besides, the SNPs (rs10041520, rs4582314 and rs4634384) have strong linkage disequilibrium (r^2^>0.8) with rs33388 are located on the promoters of *NR3C1* which were strongly modified by histone H3K27Ac, therefore, these alterations might result in aberrant transcription of *NR3C1* through altering the activity of these promoters. Moreover, refer to study of Westra *et al*., rs258763 and rs853175 which have strong linkage disequilibrium (r^2^>0.8) with rs33388 show cis-eQTL with *NR3C1* expression in whole blood [32].

The SNP rs4912913 is located 2.9kb far from 5′ of *NR3C1* and the motif is predicted to be bound by transcription factor by SNPinfo. The SNPs have strong linkage disequilibrium (r^2^>0.8) with rs4912913 are located on the enhancers which were strongly modified by histone H3KMe1 and H3K27Ac, therefore, these alterations might result in aberrant transcription of *NR3C1* through change the activation of these enhancers.

As a nuclear hormone receptor super family of transcription factors [19], NR3C1 is a main regulator of glucocorticoid hormone effects, leading to altered glucocorticoid expression in tissues [20–23]. Glucocorticoids play as a natural defensive factors in maintaining the integrity of the gastric mucosa and might operate to attenuate NSAID-induced gastropathy [30]. The above results imply that SNPs rs33388 and rs12521436 might influence gastric cancer development through regulation of *NR3C1* expression.

In lung cancer and childhood acute lymphoblastic leukemia, *NR3C1* genetic variants were significantly associated with the cancer susceptibility [33, 34]. We clearly showed that the three SNPs were significantly associated with GC risk in Chinese populations and they may be useful as genetic markers in risk prediction of gastric cancer in Chinese. However several limitations of our study need to be addressed. First, though aberrant DNA methylation in the promoter regions of *NR3C1* is well-defined epigenetic hallmark in gastric cancer [35, 36]. We did not evaluate effects of SNPs on the methylation levels of *NR3C1* promoters. Second, we could not access additional independent samples to confirm our results and it would be better if we could confirm our results using some functional assays.

## MATERIALS AND METHODS

### Study subjects

This study was approved by the institutional review board of Nanjing Medical University and informed consent has obtained from all participants. Patients with gastric cancer were consecutively recruited in cities including Yangzhong and Yixing from January 2004 to July 2005, and cities of Yangzhou from October 2006 to June 2010 and Nanjing from October 2006 to December 2011 in Jiangsu Province, Eastern China. All of the gastric cancer cases were histopathologically confirmed, and those having a history of any cancer or having undergone radiotherapy or chemotherapy were excluded. As a result, 1,113 newly diagnosed gastric cancer cases were included in this study. A total of 1,848 controls were randomly selected from a pool of more than 40,000 cancer-free individuals who participated in the community-based screening program for non-infectious diseases conducted in Jiangsu province. After signing informed consent, each subject was face-to-face interviewed with a standard questionnaire including information on demographic data and related risk factors, such as tobacco smoking and alcohol drinking. After interview, each participant donated ∼5 ml venous blood sample. Finally, 1,113 incident GC cases and 1,848 frequency-matched controls were included in this study. Individuals who smoked at least once a day for >1 year were defined as smokers and those who drank twice or more per week for at least 1 year were considered drinkers. The details for the characteristics of study subjects were shown in Supplementary Table 2. This study was approved by the institutional review board of Nanjing Medical University.

### *NR3C1* polymorphism selection and genotyping assays

For *NR3C1*, we used public HapMap SNP database to search SNPs that localized within gene regions (including 10kb up-stream region of *NR3C1*), with minor allele frequency (MAF) ≥0.05 in Chinese Han population (CHB). Then, a web-based analysis tool was used to predict the function of these SNPs (https://snpinfo.niehs.nih.gov/snpinfo/snpfunc.html). After that, we conducted a SNPs tagging analysis using Haploview4.2 software to get tagging SNPs of each gene, all the predicted functional SNPs were included as tagging SNPs in this step. As a result, 4 SNPs were found in *NR3C1* (Supplementary Table 1). Each of these SNPs has a MAF of at least 5% in Chinese population. None of these SNPs were in strong linkage disequilibrium (r^2^>0.8), and thus selected to be genotyped in this study (Supplementary Figure 2 and Supplementary Table 6).

All methods and experimental protocols were approved by Jiangsu Key Lab of Cancer Biomarkers, Prevention and Treatment, Collaborative Innovation Center for Cancer Personalized Medicine, and carried out in accordance with the approved guidelines. Genomic DNA was isolated from leukocyte pellets of venous blood by proteinase K digestion and followed by phenolchloroform extraction. The genotyping was performed by Illumina Infinium® BeadChip (Illumina Inc.) without knowing the subjects' case or control status. Genotype calling was performed using the GenTrain version 1.0 clustering algorithm in GenomeStudioV2011.1 (Illumina). All SNPs were successfully genotyped with call rates are 100%.

### Statistical and bioinformatics analysis

The chi-square test was used to evaluate distribution differences of demographic characteristics, selected variables and genotypes between cases and controls. HWE between SNPs was tested using a goodness-of-fit chi-square test among the control subjects. Logistic regression analyses were employed to test the association between genetic variants and gastric cancer risk, to evaluate odds ratios (ORs) and 95% confidence intervals (CIs) with an adjustment for age, sex, smoking, drinking status and the top ten principal components. The trend test was performed using logistic regression analysis. To evaluate the associations between the tag SNPs and gastric cancer risk, unconditional logistic regression models were used to calculate ORs and their 95% CIs adjusted for age, sex, smoking status, drinking status and the top ten principal components. The detailed information about the top ten principal components could be obtained from our previous study [33]. In brief, the population structure was evaluated using principal component analysis using EIGENSOFT4.2 based on 4,861 autosomal scaffold markers included in the exome array. To examine the differences between subgroups, the chi-square-based Q-test was used to test the heterogeneity of effect sizes (ORs and 95% CIs) derived from corresponding subgroups. PHASE v2.1 was used to estimate the haplotype for each individual based on the observed genotypes. All statistical analyses were performed with R3.3.2 and Plink1.07.

Expression abundance of *NR3C1* was downloaded from TCGA database: https://confluence.broadinstitute.org/display/GDAC/Dashboard-Stddata (July, 2014), referred to Expectation-Maximization (RSEM) normalized read counts including tumor tissues and corresponding adjacent normal tissues. Then, we computed the mRNA expression levels using the RSEM data and finally used the integrated data to explore the expression difference of specific gene (238 tumor tissues and 33 corresponding adjacent normal tissues). Moreover, refer to study of Miao *et al*., functional annotation for SNPs was performed through HaploRegV3 (http://www.broadinstitute.org/mammals/haploreg/haploreg.php) and ENCODE database [37, 38].

## SUPPLEMENTARY FIGURES AND TABLES





## Abbreviations

*NR3C1*: Nuclear receptor subfamily 3 group C member 1
SNPs: Single nucleotide polymorphisms
GC: Gastric cancer
5YSR: 5 year survival rate
GWAS: Genome-wide association studies
HCC: Hepatocellular carcinoma
NSAID: Nonsteroidal anti-inflammatory drug
HWE: Hardy-Weinberg equilibrium
TFBS: Transcription factor binding sites
MAF: Minor allele frequency
CHB: Chinese Han population
ORs: Odds ratios
CIs: Confidence intervals
RSEM: Expectation-Maximization normalized read counts

## References

[1] Brenner H, Rothenbacher D, Arndt V (2009). Epidemiology of stomach cancer. Methods Mol Biol.

[2] Wang J, Zhang J, Zhou C, Chen L, Yu Q (2014). An Insertion/Deletion Polymorphism Within the Proximal Promoter of EGLN2 Is Associated With Susceptibility for Gastric Cancer in the Chinese Population. Genet Test Mol Biomarkers.

[3] Bertuccio P, Chatenoud L, Levi F, Praud D, Ferlay J, Negri E, Malvezzi M, La Vecchia C (2009). Recent patterns in gastric cancer: a global overview. Int J Cancer.

[4] Chen W, Zheng R, Baade PD, Zhang S, Zeng H, Bray F, Jemal A, Yu XQ, He J (2016). Cancer statistics in China, 2015. CA Cancer J Clin.

[5] Helicobacter and Cancer Collaborative Group (2001). Gastric cancer and Helicobacter pylori: a combined analysis of 12 case control studies nested within prospective cohorts. Gut.

[6] Smith GD, Hart C, Blane D, Hole D (1998). Adverse socioeconomic conditions in childhood and cause specific adult mortality: prospective observational study. BMJ.

[7] Saha AK, Maitra S, Hazra SC (2013). Epidemiology of gastric cancer in the gangetic areas of west bengal. ISRN Gastroenterol.

[8] Stoicov C, Saffari R, Cai X, Hasyagar C, Houghton J (2004). Molecular biology of gastric cancer: Helicobacter infection and gastric adenocarcinoma: bacterial and host factors responsible for altered growth signaling. Gene.

[9] Ghasemi-Kebria F, Ghaemi E, Azadfar S, Roshandel G (2013). Epidemiology of Helicobacter pylori infection among Iranian children. Arab J Gastroenterol.

[10] Chen J, Bu XL, Wang QY, Hu PJ, Chen MH (2007). Decreasing seroprevalence of Helicobacter pylori infection during 1993-2003 in Guangzhou, southern China. Helicobacter.

[11] Yang Q, Shao Y, Shi J, Qu Y, Wu K, Dang S, Shi B, Hou P (2014). Concomitant PIK3CA amplification and RASSF1A or PAX6 hypermethylation predict worse survival in gastric cancer. Clin Biochem.

[12] Guang W, Czinn SJ, Blanchard TG, Kim KC, Lillehoj EP (2014). Genetic regulation of MUC1 expression by Helicobacter pylori in gastric cancer cells. Biochem Biophys Res Commun.

[13] Dai N, Zheng M, Wang C, Ji Y, Du J, Zhu C, He Y, Zhu M, Zhu X, Sun M, Dai J, Ma H, Chen J (2014). Genetic variants at 8q24 are associated with the risk of esophageal squamous cell carcinoma in a Chinese population. Cancer Sci.

[14] Song HR, Kim HN, Kweon SS, Choi JS, Shim HJ, Cho SH, Chung IJ, Park YK, Kim SH, Choi YD, Joo KW, Shin MH (2014). Common genetic variants at 1q22 and 10q23 and gastric cancer susceptibility in a Korean population. Tumour Biol.

[15] Saeki N, Ono H, Sakamoto H, Yoshida T (2013). Genetic factors related to gastric cancer susceptibility identified using a genome-wide association study. Cancer Sci.

[16] Shi Y, Hu Z, Wu C, Dai J, Li H, Dong J, Wang M, Miao X, Zhou Y, Lu F, Zhang H, Hu L, Jiang Y (2011). A genome-wide association study identifies new susceptibility loci for non-cardia gastric cancer at 3q13.31 and 5p13.1. Nat Genet.

[17] Mueller KM, Themanns M, Friedbichler K, Kornfeld JW, Esterbauer H, Tuckermann JP, Moriggl R (2012). Hepatic growth hormone and glucocorticoid receptor signaling in body growth, steatosis and metabolic liver cancer development. Mol Cell Endocrinol.

[18] Vilasco M, Communal L, Mourra N, Courtin A, Forgez P, Gompel A (2011). Glucocorticoid receptor and breast cancer. Breast Cancer Res Treat.

[19] Sanchez-Vega B, Gandhi V (2009). Glucocorticoid resistance in a multiple myeloma cell line is regulated by a transcription elongation block in the glucocorticoid receptor gene (NR3C1). Br J Haematol.

[20] Adcock IM, Nasuhara Y, Stevens DA, Barnes PJ (1999). Ligand-induced differentiation of glucocorticoid receptor (GR) trans-repression and transactivation: preferential targetting of NF-kappaB and lack of I-kappaB involvement. Br J Pharmacol.

[21] Cairns C, Cairns W, Okret S (1993). Inhibition of gene expression by steroid hormone receptors via a negative glucocorticoid response element: evidence for the involvement of DNA-binding and agonistic effects of the antiglucocorticoid/antiprogestin RU486. DNA Cell Biol.

[22] Yamamoto KR, Alberts BM (1976). Steroid receptors: elements for modulation of eukaryotic transcription. Annu Rev Biochem.

[23] Drouin J, Charron J, Gagner JP, Jeannotte L, Nemer M, Plante RK, Wrange O (1987). Pro-opiomelanocortin gene: a model for negative regulation of transcription by glucocorticoids. J Cell Biochem.

[24] Ahlquist T, Lind GE, Costa VL, Meling GI, Vatn M, Hoff GS, Rognum TO, Skotheim RI, Thiis-Evensen E, Lothe RA (2008). Gene methylation profiles of normal mucosa, and benign and malignant colorectal tumors identify early onset markers. Mol Cancer.

[25] Lind GE, Kleivi K, Meling GI, Teixeira MR, Thiis-Evensen E, Rognum TO, Lothe RA (2006). ADAMTS1, CRABP1, and NR3C1 identified as epigenetically deregulated genes in colorectal tumorigenesis. Cell Oncol.

[26] Tacon LJ, Soon PS, Gill AJ, Chou AS, Clarkson A, Botling J, Stalberg PL, Skogseid BM, Robinson BG, Sidhu SB, Clifton-Bligh RJ (2009). The glucocorticoid receptor is overexpressed in malignant adrenocortical tumors. J Clin Endocrinol Metab.

[27] Pan D, Kocherginsky M, Conzen SD (2011). Activation of the glucocorticoid receptor is associated with poor prognosis in estrogen receptor-negative breast cancer. Cancer Res.

[28] Chang W, Ma L, Lin L, Gu L, Liu X, Cai H, Yu Y, Tan X, Zhai Y, Xu X, Zhang M, Wu L, Zhang H (2009). Identification of novel hub genes associated with liver metastasis of gastric cancer. Int J Cancer.

[29] Qu Y, Dang S, Hou P (2013). Gene methylation in gastric cancer. Clin Chim Acta.

[30] Filaretova L (2013). Gastroprotective role of glucocorticoids during NSAID-induced gastropathy. Curr Pharm Des.

[31] Baghdasaryan A, Chiba P, Trauner M (2013). Clinical application of transcriptional activators of bile salt transporters. Mol Aspects Med.

[32] Westra HJ, Peters MJ, Esko T, Yaghootkar H, Schurmann C, Kettunen J, Christiansen MW, Fairfax BP, Schramm K, Powell JE, Zhernakova A, Zhernakova DV, Veldink JH (2013). Systematic identification of trans eQTLs as putative drivers of known disease associations. Nat Genet.

[33] Zhu M, Yan C, Ren C, Huang X, Zhu X, Gu H, Wang M, Wang S, Gao Y, Ji Y, Miao X, Yang M, Chen J (2017). Exome Array Analysis Identifies Variants in SPOCD1 and BTN3A2 That Affect Risk for Gastric Cancer. Gastroenterology.

[34] Kaymak Cihan M, Karabulut HG, Yurur Kutlay N, Ilgin Ruhi H, Tukun A, Olcay L (2017). Association Between N363S and BclI Polymorphisms of the Glucocorticoid Receptor Gene (NR3C1) and Glucocorticoid Side Effects During Childhood Acute Lymphoblastic Leukemia Treatment. Turk J Haematol.

[35] Kang GH, Lee S, Cho NY, Gandamihardja T, Long TI, Weisenberger DJ, Campan M, Laird PW (2008). DNA methylation profiles of gastric carcinoma characterized by quantitative DNA methylation analysis. Lab Invest.

[36] Pascussi JM, Dvorak Z, Gerbal-Chaloin S, Assenat E, Maurel P, Vilarem MJ (2003). Pathophysiological factors affecting CAR gene expression. Drug Metab Rev.

[37] Li J, Zou L, Zhou Y, Li L, Zhu Y, Yang Y, Gong Y, Lou J, Ke J, Zhang Y, Tian J, Zou D, Peng X (2017). A low-frequency variant in SMAD7 modulates TGF-beta signaling and confers risk for colorectal cancer in Chinese population. Mol Carcinog.

[38] Lou J, Gong J, Ke J, Tian J, Zhang Y, Li J, Yang Y, Zhu Y, Gong Y, Li L, Chang J, Zhong R, Miao X (2017). A functional polymorphism located at transcription factor binding sites, rs6695837 near LAMC1 gene, confers risk of colorectal cancer in Chinese populations. Carcinogenesis.

