# Supplementary material for: Functional polymorphisms in NR3C1 are associated with gastric cancer risk in Chinese population
Source: Oncotarget. 2017 Oct 27;8(62):105312–9. doi: 10.18632/oncotarget.22172 (PMC5739640; doi:10.18632/oncotarget.22172)
Supplement: Supplementary file 1 [file oncotarget-08-105312-s001.pdf]

Functional polymorphisms in *NR3C1* are associated with gastric cancer risk in Chinese population

SUPPLEMENTARY MATERIALS

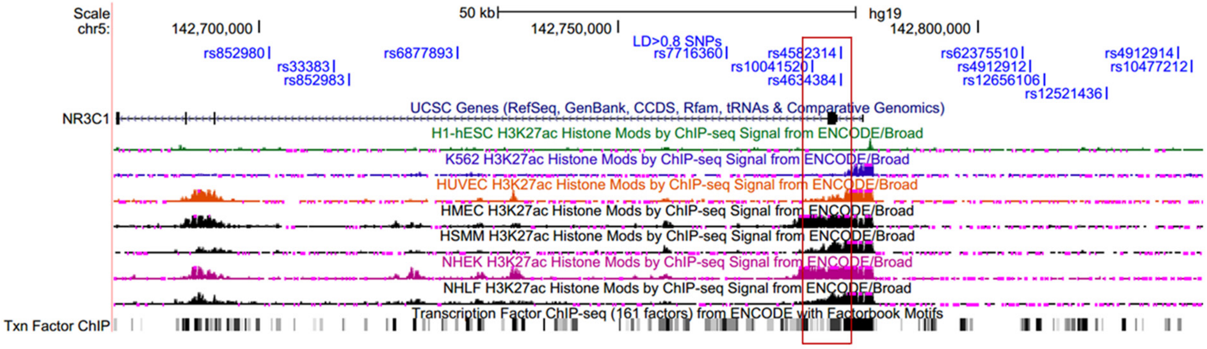

Supplementary Figure 1: SNPs have strong linkage disequilibrium with the rs33388 in ENCODE project located on the promoter of *NR3C1*. The SNPs (rs10041520, rs4582314 and rs4634384) have strong linkage disequilibrium (r^2>0.8) with rs33388 are located on the promoters of *NR3C1* which were strongly modified by histone H3K27Ac. These regions were enriched with transcription binding sites, especially locations of rs4582314 and rs4634384 in ENCODE database.

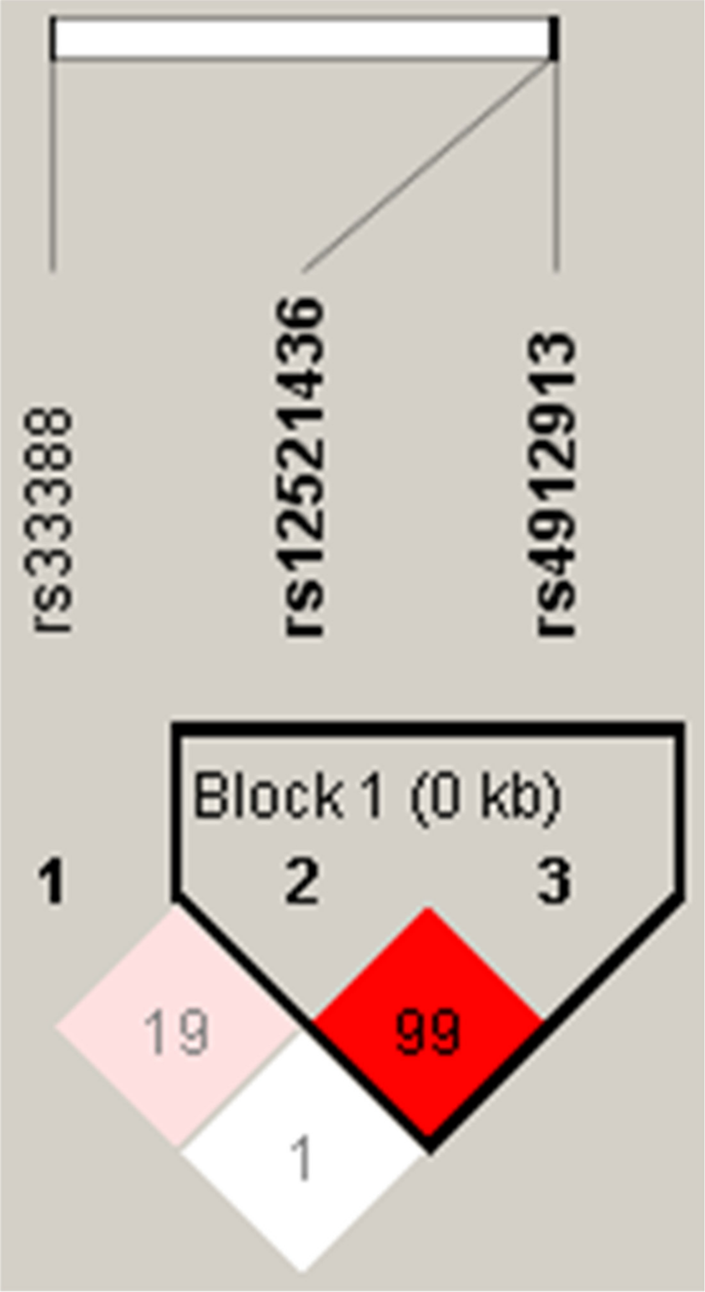

Supplementary Figure 2: Linkage disequilibrium blocks of the three SNPs were created based on the default algorithm is taken from Gabriel et al, Science, 2002, using HaploView software 4.2. The value of D' of each SNPs pair is shown in the crossing areas.

Supplementary Table 1: Basic information of four selected SNPs in *NR3C1*

SNP	Location	Major/Minor allele	MAF	Call rate	HWE[a]	TFBS[b]	Splicing ESE/ESS[b]	Nearby Gene	*P* value[c]	FDR-*P* value[d]
rs4912913	5' near	A/G	0.32	100%	0.62	Y	--	LOC729123	5.75×10^{-3}	1.15×10^{-2}
rs6194	intron 12	G/A	0.07	100%	0.59	--	Y	NR3C1	0.40	0.40
rs12521436	intron 15	G/A	0.43	100%	0.11	Y	--	LOC729123	3.12×10^{-3}	1.15×10^{-2}
rs33388	intron 15	T/A	0.23	100%	0.18	Y	--	NR3C1	1.64×10^{-2}	2.18×10^{-2}

[a] HWE: Hardy–Weinberg equilibrium. The *P* value for HWE was computed using the genotype data of controls;
[b] Functional annotation of 4 selected SNPs with SNPinfo (http://snpinfo.niehs.nih.gov/cgi-bin/snpinfo/snpfunc.cgi);
[c] The *P* value was computed using Plink1.07.
[d] The *P* value was adjusted by FDR using Plink1.07.

Supplementary Table 2: Characteristics of subjects included this study

Variable	Case (n=1,113)	Control(n=1848)	*P* value
	N (%)	N (%)	
**Age(mean±sd)**	61.02±10.51	60.76±12.05	0.544[a]
<60	466(41.87)	827(44.75)	0.135[b]
≥60	647(58.13)	1021(55.25)	
**Sex**			
Male	839 (75.38)	1343(72.67)	0.115[b]
Female	274 (24.62)	505(27.33)	
**Smoking status**			
Never	578(51.93)	874(47.29)	0.016[b]
Ever	535(48.07)	974(52.71)	
**Drinking status**			
Never	663(59.57)	1115(60.34)	0.709[b]
Ever	450(40.43)	733(39.66)	

[a] Welch's t-test was applied for the unequal variances;
[b] Two-sided Chi-square test.

Supplementary Table 3: Stratified analyses of associations between rs33388, rs12521436, rs4912913 and gastric cancer risk

Variables	rs33388(*NR3C1*, T/A)			rs12521436 (*NR3C1*, G/A)			rs4912913(*NR3C1*, A/G)		
	OR (95%CI)[a]	*P*[a]	*P*[b]	OR (95%CI)[a]	*P*[a]	*P*[b]	OR (95%CI)[a]	*P*[a]	*P*[b]
**Age (years)**									
<60	0.81(0.65-0.99)	0.043	0.552	0.99(0.84-1.18)	0.936	0.016	0.99(0.83-1.19)	0.945	0.012
≥60	0.88(0.74-1.05)	0.159		0.75(0.65-0.87)	1.35×10^{-4}		1.35(1.15-1.59)	1.96×10^{-4}	
**Sex**									
Male	0.91(0.78-1.07)	0.250	0.020	0.87(0.76-0.99)	0.038	0.277	1.16(1.00-1.33)	0.043	0.265
Female	0.61(0.45-0.83)	1.37×10^{-3}		0.75(0.59-0.95)	0.016		1.36(1.07-1.73)	0.013	
**Smoking status**									
Never	0.87(0.72-1.06)	0.176	0.803	0.79(0.67-0.93)	3.82×10^{-3}	0.263	1.24(1.04-1.47)	0.014	0.741
Ever	0.84(0.69-1.02)	0.075		0.90(0.76-1.06)	0.203		1.19(1.00-1.42)	0.051	
**Drinking status**									
Never	0.84(0.70-0.99)	0.043	0.620	0.82(0.71-0.95)	6.95×10^{-3}	0.544	1.24(1.07-1.45)	5.70×10^{-3}	0.454
Ever	0.90(0.73-1.11)	0.327		0.88(0.74-1.05)	0.165		1.13(0.94-1.36)	0.203	

[a] Derived from additive model using logistic regression analysis with an adjustment for age, sex, smoking, drinking status and the top ten principal components;
[b] *P* for heterogeneity test based on χ^2-based Q test.

Supplementary Table 4: The interaction of rs12521436 and rs4912913 with age on gastric cancer risk

Age	rs12521436			Adjusted OR (95%CI)	P^a	rs4912913			Adjusted OR (95%CI)	P^a
	Genotype	Case	Control			Genotype	Case	Control		
<60	GG	147	269	1.00		AA	218	392	1.00	
<60	GA	241	389	1.18(0.90-1.54)	0.230	AG	202	352	0.98(0.76-1.26)	0.860
<60	AA	78	169	0.92(0.65-1.30)	0.634	GG	46	83	1.01(0.67-1.52)	0.971
≥60	GG	248	306	1.50(1.14-1.98)	3.39×10^{-3}	AA	267	498	0.93(0.73-1.17)	0.514
≥60	GA	301	492	1.15(0.89-1.49)	0.282	AG	297	440	1.20(0.95-1.51)	0.124
≥60	AA	98	223	0.85(0.61-1.17)	0.32	GG	83	83	1.74(1.21-2.49)	2.80×10^{-3}
	P for multiplicative interaction				0.018	*P* for multiplicative interaction				0.013

[a] *P* value of interaction analysis on gastric cancer risk with adjustment for sex, smoking, drinking status and the top ten principal components.

Supplementary Table 5: Functional annotation for rs33388, rs12521436, rs4912913 and those with strong linkage disequilibrium with these SNPs in HaploRegV3

See Supplementary file 1

Supplementary Table 6: Linkage disequilibrium (r^2 / D’) information of the three SNPs

SNPs	rs33388	rs12521436	rs4912913
rs33388	-	0.01(0.19)	0(0.01)
rs12521436	0.01(0.19)	-	0.35(0.99)
rs4912913	0(0.01)	0.35(0.99)	-
