# Supplementary material for: Functional polymorphisms in NR3C1 are associated with gastric cancer risk in Chinese population
Source: Oncotarget. 2017 Oct 27;8(62):105312–9. doi: 10.18632/oncotarget.22172 (PMC5739640; doi:10.18632/oncotarget.22172)
Supplement: Supplementary file 2 [file oncotarget-08-105312-s002.docx]

**Supplementary Table 5:** Functional annotation for rs33388, rs12521436, rs4912913 and those with strong linkage disequilibrium with these SNPs in HaploRegV3

| SNPs | LD(r²) | Ref | Alt | ASN freq | Promoter | Enhancer | Proteins bound | Motifs changed | GENCODE genes |
| --- | --- | --- | --- | --- | --- | --- | --- | --- | --- |
| [rs853180](http://www.broadinstitute.org/mammals/haploreg/detail_v2.php?query=&id=rs853180) | 0.89 | A | G | 0.25 | - | 6 cell types | - | 5 altered motifs | Intergenic |
| [rs853183](http://www.broadinstitute.org/mammals/haploreg/detail_v2.php?query=&id=rs853183) | 1.00 | C | A | 0.24 | - | 4 cell types | CEBPB,STAT3 | AP-3,Nkx2,TATA | Intergenic |
| [rs258763](http://www.broadinstitute.org/mammals/haploreg/detail_v2.php?query=&id=rs258763) | 1.00 | T | A | 0.24 | - | - | - | Hbp1,PRDM1 | Intergenic |
| [rs258747](http://www.broadinstitute.org/mammals/haploreg/detail_v2.php?query=&id=rs258747) | 0.84 | A | G | 0.28 | - | - | - | DMRT1,Pou5f1,Sox | Intergenic |
| [rs6191](http://www.broadinstitute.org/mammals/haploreg/detail_v2.php?query=&id=rs6191) | 0.95 | C | A | 0.23 | - | - | - | FAC1,Foxp1,RREB-1 | NR3C1 |
| [rs852976](http://www.broadinstitute.org/mammals/haploreg/detail_v2.php?query=&id=rs852976) | 1.00 | T | C | 0.76 | - | - | - | - | NR3C1 |
| [rs33388](http://www.broadinstitute.org/mammals/haploreg/detail_v2.php?query=&id=rs33388) | 1.00 | A | T | 0.76 | - | 4 cell types | - | Cphx,RXRA | NR3C1 |
| [rs852980](http://www.broadinstitute.org/mammals/haploreg/detail_v2.php?query=&id=rs852980) | 0.97 | G | C | 0.76 | - | - | - | Gfi1 | NR3C1 |
| [rs33383](http://www.broadinstitute.org/mammals/haploreg/detail_v2.php?query=&id=rs33383) | 0.98 | C | T | 0.76 | - | NHLF | - | 4 altered motifs | NR3C1 |
| [rs852983](http://www.broadinstitute.org/mammals/haploreg/detail_v2.php?query=&id=rs852983) | 0.95 | G | A | 0.76 | - | - | - | 5 altered motifs | NR3C1 |
| [rs6877893](http://www.broadinstitute.org/mammals/haploreg/detail_v2.php?query=&id=rs6877893) | 0.95 | G | A | 0.76 | - | GM12878, HepG2 | - | AIRE | NR3C1 |
| [rs7716360](http://www.broadinstitute.org/mammals/haploreg/detail_v2.php?query=&id=rs7716360) | 0.94 | T | C | 0.76 | - | - | 4 bound proteins | ERalpha-a,Esr2,GR | NR3C1 |
| [rs10041520](http://www.broadinstitute.org/mammals/haploreg/detail_v2.php?query=&id=rs10041520) | 0.87 | T | C | 0.76 | 6 cell types | K562, Huvec | - | Bbx,Foxj2,Sox | NR3C1 |
| [rs4582314](http://www.broadinstitute.org/mammals/haploreg/detail_v2.php?query=&id=rs4582314) | 0.85 | C | A | 0.77 | 6 cell types | Huvec, H1, HSMM | - | Pbx3,SP2 | NR3C1 |
| [rs4634384](http://www.broadinstitute.org/mammals/haploreg/detail_v2.php?query=&id=rs4634384) | 0.85 | C | T | 0.77 | 6 cell types | Huvec, H1, HSMM | - | AIRE,Pbx3,SP2 | NR3C1 |
| [rs62375510](http://www.broadinstitute.org/mammals/haploreg/detail_v2.php?query=&id=rs62375510) | 0.84 | G | A | 0.43 | - | GM12878 | - | GATA | NR3C1 |
| rs4912912 | 0.87 | T | C | 0.44 | - | GM12878 | YY1,NFKB | 10 altered motifs | NR3C1 |
| [rs12656106](http://www.broadinstitute.org/mammals/haploreg/detail_v2.php?query=&id=rs12656106) | 0.88 | G | C | 0.44 | - | - | - | - | NR3C1 |
| [rs12521436](http://www.broadinstitute.org/mammals/haploreg/detail_v2.php?query=&id=rs12521436) | 1.00 | G | A | 0.45 | - | NHLF | - | Pbx-1 | Intergenic |
| [rs4912914](http://www.broadinstitute.org/mammals/haploreg/detail_v2.php?query=&id=rs4912914) | 0.89 | G | C | 0.53 | - | GM12878 | - | - | Intergenic |
| [rs10477212](http://www.broadinstitute.org/mammals/haploreg/detail_v2.php?query=&id=rs10477212) | 0.89 | T | C | 0.47 | - | - | - | HNF1,Hmbox1,NF-AT1 | Intergenic |
| [rs6864679](http://www.broadinstitute.org/mammals/haploreg/detail_v2.php?query=&id=rs6864679) | 0.84 | G | A | 0.54 | - | - | - | 7 altered motifs | Intergenic |
| [rs7703348](http://www.broadinstitute.org/mammals/haploreg/detail_v2.php?query=&id=rs7703348) | 0.84 | T | C | 0.54 | - | - | - | - | Intergenic |
| [rs7701487](http://www.broadinstitute.org/mammals/haploreg/detail_v2.php?query=&id=rs7701487) | 0.84 | T | A | 0.54 | - | NHLF | - | 5 altered motifs | Intergenic |
| [rs6871464](http://www.broadinstitute.org/mammals/haploreg/detail_v2.php?query=&id=rs6871464) | 0.84 | T | C | 0.55 | - | - | - | Mrg1::Hoxa9 | Intergenic |
| [rs13360801](http://www.broadinstitute.org/mammals/haploreg/detail_v2.php?query=&id=rs13360801) | 0.87 | G | T | 0.28 | - | - | - | - | Intergenic |
| [rs12657291](http://www.broadinstitute.org/mammals/haploreg/detail_v2.php?query=&id=rs12657291) | 0.88 | T | C | 0.28 | - | - | - | 6 altered motifs | Intergenic |
| [rs4912913](http://www.broadinstitute.org/mammals/haploreg/detail_v2.php?query=&id=rs4912913) | 1.00 | T | C | 0.3 | - | - | - | GR | Intergenic |
| [rs7722213](http://www.broadinstitute.org/mammals/haploreg/detail_v2.php?query=&id=rs7722213) | 0.86 | A | G | 0.28 | - | - | - | LUN-1 | Intergenic |
| [rs7726546](http://www.broadinstitute.org/mammals/haploreg/detail_v2.php?query=&id=rs7726546) | 0.99 | T | C | 0.3 | - | - | - | 8 altered motifs | Intergenic |
| [rs10041406](http://www.broadinstitute.org/mammals/haploreg/detail_v2.php?query=&id=rs10041406) | 0.86 | C | T | 0.28 | - | - | - | 5 altered motifs | Intergenic |
| [rs10075551](http://www.broadinstitute.org/mammals/haploreg/detail_v2.php?query=&id=rs10075551) | 0.86 | A | C | 0.28 | - | NHLF | - | BHLHE40,DEC,Myc | Intergenic |
| [rs34480443](http://www.broadinstitute.org/mammals/haploreg/detail_v2.php?query=&id=rs34480443) | 0.86 | C | T | 0.28 | - | - | - | GATA,HDAC2 | Intergenic |
| [rs56075823](http://www.broadinstitute.org/mammals/haploreg/detail_v2.php?query=&id=rs56075823) | 0.86 | A | G | 0.29 | - | - | - | Zbtb3 | Intergenic |
| [rs28581804](http://www.broadinstitute.org/mammals/haploreg/detail_v2.php?query=&id=rs28581804) | 0.80 | C | T | 0.28 | - | - | - | 5 altered motifs | Intergenic |
| [rs7720743](http://www.broadinstitute.org/mammals/haploreg/detail_v2.php?query=&id=rs7720743) | 0.88 | T | A | 0.3 | - | NHLF | - | 8 altered motifs | Intergenic |
| [rs7721059](http://www.broadinstitute.org/mammals/haploreg/detail_v2.php?query=&id=rs7721059) | 0.88 | T | C | 0.3 | - | NHLF | - | Pou2f2,STAT | Intergenic |
| [rs12652704](http://www.broadinstitute.org/mammals/haploreg/detail_v2.php?query=&id=rs12652704) | 0.88 | A | G | 0.3 | - | - | - | RBP-Jkappa | Intergenic |
| [rs4128753](http://www.broadinstitute.org/mammals/haploreg/detail_v2.php?query=&id=rs4128753) | 0.80 | G | A | 0.28 | - | GM12878 | - | ATF3,Ets | Intergenic |
| [rs4128755](http://www.broadinstitute.org/mammals/haploreg/detail_v2.php?query=&id=rs4128755) | 0.80 | C | T | 0.28 | - | GM12878 | - | 9 altered motifs | Intergenic |
| [rs34624957](http://www.broadinstitute.org/mammals/haploreg/detail_v2.php?query=&id=rs34624957) | 0.80 | C | T | 0.28 | - | GM12878 | - | Pdx1 | Intergenic |
| [rs6863190](http://www.broadinstitute.org/mammals/haploreg/detail_v2.php?query=&id=rs6863190) | 0.88 | T | G | 0.3 | - | GM12878 | EBF1 | RXRA | Intergenic |
